# Supplementary figures and images for: Comparative Proteomic Analyses of Susceptible and Resistant Maize Inbred Lines at the Stage of Enations Forming following Infection by Rice Black-Streaked Dwarf Virus
Source: Viruses. 2022 Nov 23;14(12):2604. doi: 10.3390/v14122604 (PMC9785138; doi:10.3390/v14122604)

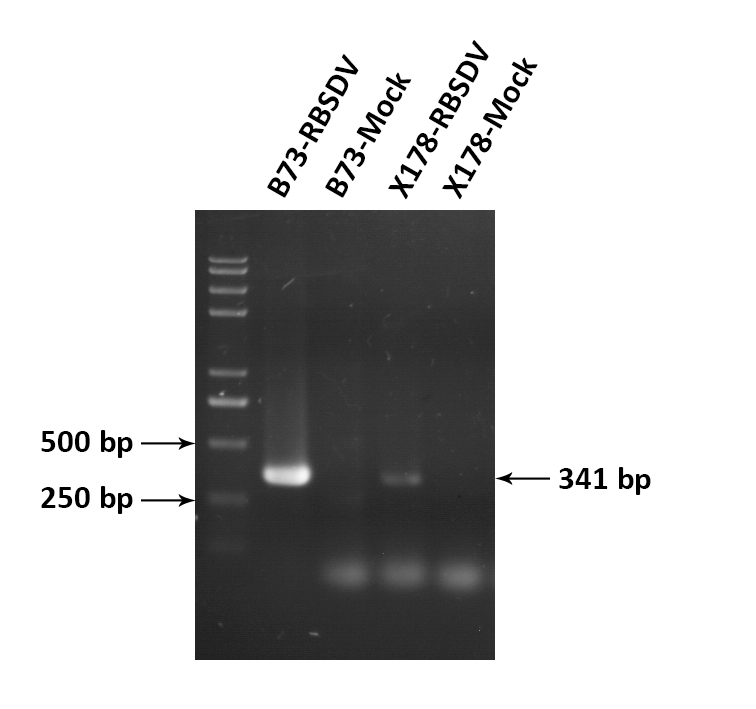

Supplement: Supplementary file 1 [file viruses-14-02604-s001.zip › Figure S1-RT-PCR detecting viral infection.jpg]

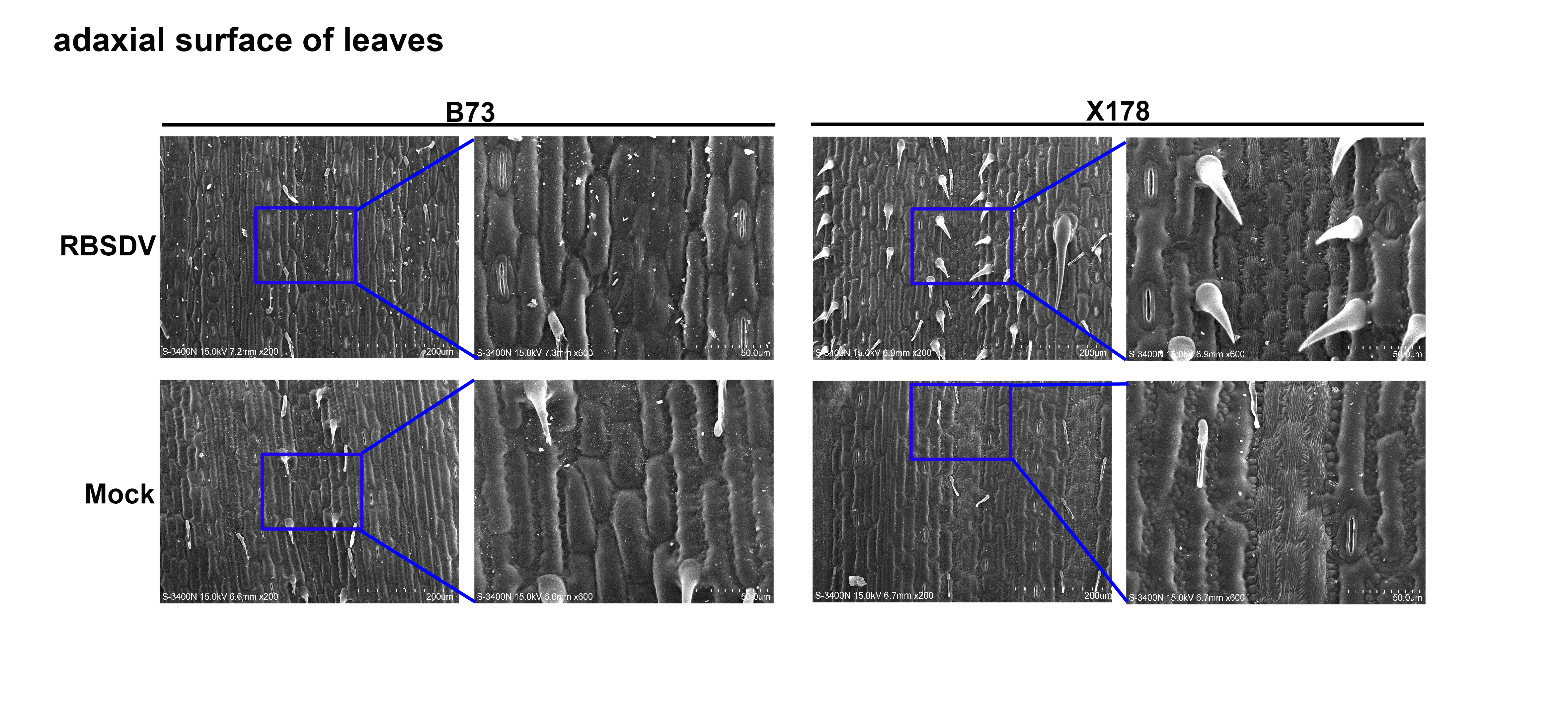

Supplement: Supplementary file 1 [file viruses-14-02604-s001.zip › Figure S2-Scanning electron micrograph of adaxial surface of leaves.jpg]
